# The Effect of Radiotherapy on Diffuse Low-Grade Gliomas Evolution: Confronting Theory with Clinical Data

**DOI:** 10.3390/jpm11080818

**Published:** 2021-08-21

**Authors:** Léo Adenis, Stéphane Plaszczynski, Basile Grammaticos, Johan Pallud, Mathilde Badoual

**Affiliations:** 1 CNRS/IN2P3, IJCLab, Université Paris-Saclay, 91405 Orsay, France; leo.adenis@outlook.fr (L.A.); grammati@paris7.jussieu.fr (B.G.); mathilde.badoual@ijclab.in2p3.fr (M.B.); 2 IJCLab, Université de Paris, 91405 Orsay, France; 3 Department of Neurosurgery, GHU Paris, Sainte-Anne Hospital, 75014 Paris, France; johanpallud@hotmail.com; 4 Université de Paris, Sorbonne Paris Cité, 75014 Paris, France; 5 Inserm, U1266, IMA-Brain, Institut de Psychiatrie et Neurosciences de Paris, 75014 Paris, France

**Keywords:** mathematical modeling, gliomas, radiotherapy, optimization, data analysis

## Abstract

Diffuse low-grade gliomas are slowly growing tumors that always recur after treatment. In this paper, we revisit the modeling of the evolution of the tumor radius before and after the radiotherapy process and propose a novel model that is simple yet biologically motivated and that remedies some shortcomings of previously proposed ones. We confront this with clinical data consisting of time series of tumor radii from 43 patient records by using a stochastic optimization technique and obtain very good fits in all cases. Since our model describes the evolution of a tumor from the very first glioma cell, it gives access to the possible age of the tumor. Using the technique of profile likelihood to extract all of the information from the data, we build confidence intervals for the tumor birth age and confirm the fact that low-grade gliomas seem to appear in the late teenage years. Moreover, an approximate analytical expression of the temporal evolution of the tumor radius allows us to explain the correlations observed in the data.

## 1. Introduction

Gliomas are tumors of the central nervous system that arise from precursors of glial cells and account for almost 80% of primary malignant brain tumors. Although relatively rare, they result in more years of life lost than any other tumor: approximately 13,000 deaths and 18,000 new cases of primary malignant brain and central nervous system tumors occur annually in the United States [1]. Historically, the tumors of the central nervous system have been classified by the World Health Organization into four grades based on their histological characteristics and on the aggressiveness of the tumor [1]: grade 1 gliomas are benign, well delineated, and can be cured by surgery. In grades 2 and above, the tumors are diffuse and, because of that, incurable. Recently, a revision of the World Health Organization classification was proposed, and it is now the isocitrate dehydrogenase (IDH) enzyme mutation status that allows one to classify these tumors in the first place [2]. In this paper, we mostly use data from patients who were recruited before 2016. The status of the IDH enzyme mutation was not assessed, so we just followed the WHO classification that was in use at the time of diagnosis and used the term of diffuse low-grade gliomas (DLGGs) for these patients’ tumors, which included low-grade astocytomas and oligodendrogliomas [3].

In high-grade gliomas, the rate of proliferation is very large, and in the center, the cells become hypoxic and, finally, necrotic. In contrast, in DLGGs, the rate of proliferation is lower, and these tumors are composed only of isolated migrating tumor cells that infiltrate the normal tissue. On a magnetic resonance imaging (MRI) scan, DLGGs present a T1 hypointense signal without contrast enhancement (since there is no angiogenesis), but also a T2-Fluid Attenuated Inversion Recovery (FLAIR) hyperintense signal [4,5]. It has been shown that tumor cells migrate well beyond the limits of the tumor’s hyperintense area on T2-FLAIR-weighted MRI scans [6,7].

Though DLGGs are associated with an extended life expectancy compared to higher-grade gliomas, they represent a real public health issue because patients are often young (between 20 and 40 years old) with a previously normal social and professional life. DLGGs grow slowly, but their invasive features are responsible for their unavoidable recurrence, even after oncological treatments [8].

Treatments primarily consist of surgery when possible. Chemotherapy and then radiotherapy are proposed for progressive residual tumors and at tumor progression. However, despite technical progress in imaging techniques and therapeutic management, treatment only confers a modest improvement in overall survival [9,10,11,12]. However, even worse, all low-grade lesions eventually evolve into higher-grade malignant tumors when neoangiogenesis is triggered [13]. For DLGGs, the goals of radiotherapy (RT) are to control tumor growth, improve progression-free survival and patient quality of life by reducing the risk of seizures, and delay anaplastic transformation [14].

Several aspects of DLGGs have already been the objects of models, from their origin [15] to their natural evolution [11,16], their response to treatments (in particular, with RT [17,18,19,20,21]), and their anaplastic transformation [22].

We will now focus on the previous models of DLGGs under RT. In [17], two populations of cells are defined: one that is quiescent and another one that proliferates. RT damages the cells of the proliferating population, thus transforming them into quiescent cells. The model is based on ordinary differential equations and does not include any spatial structures. However, in the case of gliomas, a spatial structure is essential in a model, since a key feature of DLGGs is their capacity to invade surrounding normal tissue. In [23], the authors use the diffusion–proliferation model with a term for cell death due to RT (present only while the therapy lasts), and they applied it to high-grade gliomas. However, this model is not adequate for low-grade gliomas because it cannot account for the most striking feature of the clinical follow up, i.e., the reduction of the tumor radius, which lasts much longer than the treatment with RT itself. In [18], our group proposed a diffusion–proliferation model coupled with the production of edema by tumor cells. We successfully fitted 29 follow-ups of patients. However, even though this model is the closest to the biological characteristics of DLGGs, it involves five parameters, including the two parameters for the edema dynamics. These two parameters are unknown and cannot be easily experimentally measured. Without any estimation of their values to with which to compare, it is difficult to validate the model and make predictions. In [19], the authors developed a model based on a diffusion–proliferation model that involves two cell populations, one damaged by RT and one that is not damaged, similarly to that in [17]. The advantage of this model is that it contains a spatial structure and also allows a slow decrease in the tumor radius after the end of the RT treatment. The authors used the model to study the impact of a fractionation of the RT treatment [20,24]. However, these studies are theoretical, and the model was not applied to real clinical data.

In this article, we develop a simple biophysical model of DLGG evolution based on the diffusion–proliferation model with the addition of the effect of RT and confront it with clinical data from a large number (43) of patients. We use state-of-the art analysis techniques to adjust the model and show that it is possible to get an excellent agreement between the model and the data for all of the patients. We then study the birth ages of the tumors, the parameter values, and the correlations among several observables before and after RT.

## 2. Materials and Methods

### 2.1. The Patients

We had at our disposal a set of 43 patients with DLGGs who were diagnosed at the Sainte-Anne Hospital (Paris, France) from 1989 to 2000. These patients were selected according to precise criteria that are detailed elsewhere [3]. In short, only adults with typical DLGGs (that is, no angiogenesis and, thus, no contrast enhancement on gadolinium-T1 images), available clinical and imaging follow-ups before, during, and after RT, and RT as their first oncological treatment except for stereotactic biopsies were eligible. The external conformational RT was given using the same methodology (total dose, 50.4–54 Gy; 6-week period) at 2 outside institutions. The patients had an MRI follow-up before, during, and after RT. Three tumor diameters in the axial, coronal, and sagittal planes on each MRI image with T2-weighted and FLAIR sequences were measured manually. The mean radiological tumor radius was defined as half the geometric mean of these three diameters and was measured as a function of time. The error bars for the measured mean radius were estimated by clinicians and were set to ±1 mm. From this cohort, we discarded the patients that did not have any sign of tumor regrowth at the last time point or those that had fewer than five time points in their follow-up.

### 2.2. Standard Protocol Approvals, Registration, and Patient Consent

The study received the required authorizations (IRB#1: 2021/20) from the human research institutional review board (IRB00011687). The requirement to obtain informed consent was waived according to French legislation (observational retrospective study).

### 2.3. The Model

#### 2.3.1. Free Tumor Evolution

The diffusion–proliferation model plays a special role in the modeling of the evolution fields of gliomas. It is based on a differential equation governing glioma cell density, and in its simplest form, it involves only two key phenomena (and thus two parameters): the migration (modeled as a diffusion) of the cells and their proliferation. It is the mathematical translation of the fact that the rate of change in the tumor cell density at a given position is equal to the change in the tumor cell density due to diffusion plus the rate of change in the tumor cell density due to proliferation.

This model was first proposed in 1995 [25] and has been extensively used for high-grade gliomas since then [26,27,28,29,30,31]. However, in fact, the model is more adapted to DLGGs. Despite its simplicity, this model can, in particular, reproduce an important feature of DLGG growth that has been verified with clinical data, which is that the tumor radius increases linearly with time (over large amounts time) [32,33].

The diffusion–proliferation model describes the evolution of the glioma cell density ρ as

(1) $$\frac{\partial \rho }{\partial t}=D\Delta \rho +\kappa \rho \left(1-\rho \right)$$

where $\rho \left(\vec{r},t\right)=C/C_{m}$, *C* is the glioma cell density, $C_{m}$ is the maximal cell concentration that the tissue can handle (also called the carrying capacity), *D* is the diffusion coefficient of the glioma cells, and κ is the proliferation coefficient.

A tumor is a 3D object, so it seems logical to solve Equation (1) in 3D. We do not want to enter into too many details about its precise shape for each patient, so we will assume a spherical symmetry for all tumors.

In 3D, assuming a spherical symmetry of the tumor, Equation (1) becomes:

(2) $$\frac{\partial \rho (r,t)}{\partial t}=D\left(\frac{\partial^{2}\rho \left(r,t\right)}{{\partial r}^{2}}+\frac{2}{r}\frac{\partial \rho (r,t)}{\partial r}\right)+\kappa \rho \left(r,t\right)\left(1-\rho \left(r,t\right)\right)$$

As explained in [16], when introducing an auxiliary variable u=rρ, Equation (2) takes the form:

(3) $$\frac{\partial u}{\partial t}=D\frac{\partial^{2}u}{{\partial r}^{2}}+\kappa u\left(1-\frac{u}{r}\right)$$

with u(r=0,t)=0 and $\frac{\partial \rho }{\partial r}\left(r=0,t\right)=0$.

We solve Equation (3) by discretizing it on a mesh of spatial size $\delta r=10^{-2}$ mm=10μm and with a time step $\delta t=10^{-2}$ yr, using an implicit scheme for the diffusion part and a homographic-type discretization for the logistic part.

The limit of MRI-signal abnormality (with T2-weighted or FLAIR sequences) is usually assumed to be a curve of the iso-density of glioma cells. The radius of this visible part of the tumor in MRI (usually called the “tumor radius”) is thus defined as the distance *r* to the tumor’s center, where the cell density ρ crosses a fixed threshold $\rho^{*}$. The value of this parameter $\rho^{*}$ is not precisely known, but we expect that its value, as long as it stays much smaller than 1, will not have a strong influence on our conclusions. We set $\rho^{*}=0.02$ for all of the simulations [19,27].

The initial conditions are the same for all of the simulations and correspond to the appearance of the first tumor cell: ρ(r=δr,0)=1 and ρ(r>δr,0)=0. Here, we assume that the tumor has been developing with the same proliferation and diffusion coefficients since the appearance of the first tumor cell.

#### 2.3.2. Modeling RT

Next, we turn to the modeling of the radiotherapy process itself. The action of RT on the glioma cells is modeled as an instantaneous event, since the duration of the treatment (typically 6 weeks, or 0.11 yr) is negligible compared to the mean regrowth delay after RT (1.25 yr for our patients) [3]. The origin of time is set to the time of RT.

We introduce a new model to capture the essence of what happens after the radiotherapy by adding to the free evolution Equation (1) a *time-dependent*death term:

(4) $$\frac{\partial \rho_{(}\vec{r},t)}{\partial t}=D\Delta \rho \left(\vec{r},t\right)+\left[\kappa -\kappa_{D}\left(t\right)\right]\rho \left(\vec{r},t\right)\left(1-\rho \left(\vec{r},t\right)\right).$$

The simplest way to introduce some characteristic time is to choose

(5) $$\kappa_{D}\left(t\right)=\kappa_{d}e^{-(t-t_{r})/\tau }$$

for $t>t_{r}$, where $t_{r}$ is the time of RT and $\kappa_{D}\left(t\right)=0$ for $t<t_{r}$.

To the two parameters that describe the natural evolution of the tumor (κ,D) and the two others related to the effect of RT on tumor cells ($\kappa_{d},\tau$), we add a fifth one, the tumor age *T* at the time of RT. Although not derived from physical modeling, it is an unknown of the problem that must then be determined with the others (in statistical terminology, it is a nuisance parameter.). This parameter is important because we need to ensure that *T* is always smaller than the age of the patient themselves at the time of RT.

### 2.4. Fitting Procedure

For each patient, we determine the set of parameters that best fits our data by numerically performing a multidimensional minimization of the objective function:

(6) $$\chi^{2}\left(T,D,\kappa ,\kappa_{d},\tau_{d}\right)=\sum_{i=1}^{N_{data}}{\left[R_{data}\left(t_{i}\right)-R_{mod}\left(t_{i};T,D,\kappa ,\kappa_{d},\tau_{d}\right)\right]}^{2},$$

where $R_{data}\left(t_{i}\right)$ denotes the radius measured at time $t_{i}$ and $R_{mod}$ is the theoretical value of the radius. This value $R_{mod}$ is obtained by numerically solving our theoretical model, which means that the cell concentration profile is calculated at each time, as well as by following our model equations and thresholding it at $\rho^{*}$ to obtain the radius (we recall that the error on the measurements is about 1 mm, so there is no need to rescale the residuals.).

We also add the constraint that from a radius of 15 mm, the tumor should evolve almost in the asymptotic regime. This linearity has been observed in clinical data [32] and has already been implemented in [16]. More specifically, we compute the relative difference between the velocity from the model (computed as the slope of the radius curve) and the asymptotic value $c=2\sqrt{D\kappa }$

(7) $$r_{15}=\left(\frac{dR_{mod}}{dt}\;\left(15\right)-c\right)/c,$$

and if this value exceeds 20% we add a quadratic term to the $\chi^{2}$:

(8) $$\chi_{extra}^{2}={\left(\frac{r_{15}-0.20}{0.01}\right)}^{2}.$$

Finally, to avoid aberrant values, we will use some light bounds on the possible parameter range: $0<D<10\;{\mathrm{mm}}^{2}/\mathrm{yr}$, $0<\kappa <10\;{\mathrm{yr}}^{-1}$, $0<\kappa_{d}<500\;{\mathrm{yr}}^{-1}$, and $0<\tau_{d}<50\;\mathrm{yr}$.

The 5D optimization problem from a non-analytical and non-linear equation is challenging for standard minimization procedures. These procedures often rely on the use of analytical gradients, which are not available here. After several tests, the optimization method that we chose is the *covariance matrix adaptation evolution strategy* (CMA-ES (http://cma.gforge.inria.fr (accessed on 2 February 2020)) [34]. It is a stochastic method that belongs to the class of evolutionary algorithms and is often used for challenging optimization problems. The algorithm CMA-ES proceeds as follows: At each time step, several new candidate solutions are sampled from a multivariate normal distribution, and the *N* candidate solutions that correspond to the smallest value of the objective function *f* are selected. A weighted combination of the *N* best candidate solutions is used to update the internal state variables, such as the mean of the distribution of candidates, the step size, and the covariance matrix. One advantage of this method over other evolutionary ones is that there are only a few parameters that have to be chosen: the starting point, some estimate of the associated errors (which we choose to be about 10%), and the population size, which we tuned to 50 to obtain stable results. For each patient, since the algorithm is stochastic, 10 runs are performed, and the best fit (lowest $\chi_{min}^{2}$ value) is kept. In practice, the 10 results are very similar.

## 3. Results

### 3.1. Characterization of Our Model

**Figure 1:**
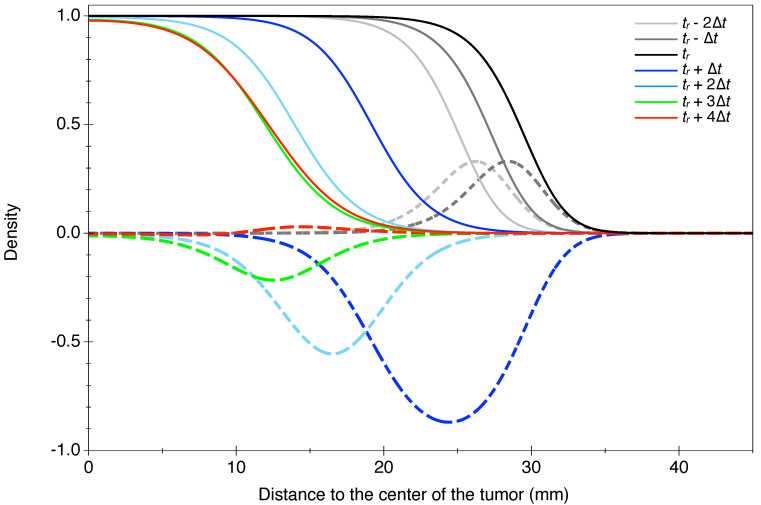
Cell density (full lines) and proliferating/killed cell density (dashed lines) profiles for different times: before (light and dark gray lines), at the time of RT (black lines), and after RT (colored lines). The time interval between two profiles is Δt=1.1 yr. The profile of proliferating/killed cells is obtained by subtracting two successive profiles. Parameters: κ=1.3 yr${}^{-1}$, D=0.8 mm${}^{2}$ yr${}^{-1}$, $\kappa_{d}=8$ yr${}^{-1}$,$\tau_{d}=2$ yr.

In Equation (4), the $\kappa -\kappa_{D}\left(t\right)$ term accounts for a net proliferation that can be positive if cells are actually created (before RT, dashed lines on Figure 1) or negative if cells are killed (after RT, colored lines). In Figure 1, one can see that before RT, the front of the profiles moves with a constant positive velocity, and the same amounts of proliferating cells are created during a given time interval (light gray, dark gray, and black profiles of proliferating cells, dashed lines). Since the center of the tumor reaches saturation, the proliferating cells are located at the border of the tumor. After RT, the front moves backwards, and the net proliferation becomes negative: Cells are killed. Since the death term has exactly the same structure as the proliferation term, cells do not die where the cell density is close to saturation—at the center of the tumor. Cells are killed at the border, and because the death parameter decreases exponentially with time, the amount of cells dying during each time interval decreases. After some time, proliferation surpasses death, and the tumor starts to regrow (see the pink profile, Figure 1).

This is different from models with two populations (damaged/undamaged cells) with constant death rates, where the density is uniformly decreased. As shown later, these models lead to a linear decrease just after RT (which is clearly not what is observed), while our model allows for an exponential-type decrease (more about the comparison in the Appendix A).

Simple analytical considerations can give insights about the early linear-versus-exponential decrease in the radius. If we assume that, at the time of RT, the asymptotic regime is reached, then the profile of the cell density is a sigmoidal curve, and the front propagates at a constant velocity:

(9) $$v=2\sqrt{D\kappa }$$

At the time of RT ($t=t_{r}$), the profile of the cell density then follows [35]:

(10) $$\rho \left(r,t_{r}\right)≃\frac{1}{1+\exp((r-r_{1/2})/\lambda )}$$

where the characteristic length is $\lambda =2\sqrt{\frac{D}{\kappa }}$ and $r_{1/2}$ is defined so that $\rho (r_{1/2},t_{r})=1/2$.

Since we are interested in the evolution of the radius, corresponding to a very low threshold of the cell density ($\rho^{*}=0.02<<1$), the profile is locally well described near $\rho^{*}$ by

(11) $$\rho \left(r,t_{r}\right)≃\exp(-(r-r_{1/2})/\lambda ).$$

Just after RT, the time during which the radius decreases before regrowth is short enough to neglect the effect of the diffusion. In this case, close to the threshold where the saturation term can be neglected, the cell density follows the equation:

(12) $$\begin{array}{cc} \frac{d\rho }{dt} & =(\kappa -\kappa_{D}\left(t\right))\rho \left(1-\rho \right)\\ & ≃(\kappa -\kappa_{D}\left(t\right))\rho \end{array}$$

The solution to this equation for $t>t_{r}$ is:

(13) $$\rho \left(r,t\right)=\rho \left(r,t_{r}\right)\exp\left(\kappa \left(t-t_{r}\right)-\int_{t_{r}}^{t}\kappa_{d}dt\right).$$

So, after RT, the cell density close to the threshold (at large *r*) can be rewritten as:

(14) $$\rho \left(r,t\right)=\exp\left(-\left(r-r_{1/2}\right)/\lambda +\kappa \left(t-t_{r}\right)-\int_{t_{r}}^{t}\kappa_{d}dt\right).$$

Setting $\rho =\rho^{*}$, one finds the evolution of the radius at the threshold:

(15) $$r\left(t\right)=r_{1/2}+\lambda \left(\kappa \left(t-t_{r}\right)-\int_{t_{r}}^{t}\kappa_{d}dt-\ln\left(\rho^{*}\right)\right).$$

If the death term is constant, then $\int_{t_{r}}^{t}\kappa_{d}dt=\kappa_{d}\left(t-t_{r}\right)$, and the radius just after RT varies linearly with a constant velocity: $\lambda (\kappa -\kappa_{d})$. On the other hand, if $\kappa_{D}\left(t\right)$ is an exponential function, we obtain:

(16) $$r\left(t\right)=r_{1/2}+\lambda \left(\kappa \left(t-t_{r}\right)+\kappa_{d}\tau_{d}\left(e^{-\left(t-t_{r}\right)/\tau_{d}}-1\right)-\ln\left(\rho^{*}\right)\right).$$

We can write this equation in a more simple way by reintroducing the effective velocity *v* Equation (9) and defining $r_{r}=r\left(t_{r}\right)$ as the radius of the tumor at the time of RT. After RT (for $t>t_{r}$), the equation of evolution of the tumor radius is:

(17) $$r\left(t\right)=r_{r}-v\tau_{d}\frac{\kappa_{d}}{\kappa }\left(1-e^{-\left(t-t_{r}\right)/\tau_{d}}\right)+v\left(t-t_{r}\right)$$

This evolution is similar to that in a two-population model where the damaged population decreases exponentially with some characteristic time $\tau_{d}$ and amplitude $v\tau_{d}\frac{\kappa_{dm}}{\kappa }$, and the undamaged population still grows linearly with the asymptotic speed vt.

Finally, we stress that this is an approximate description aiming at capturing the gross features of the evolution of the radius. In the following, the exact Equation (4) is numerically solved.

### 3.2. Best Fits

**Figure 2:**
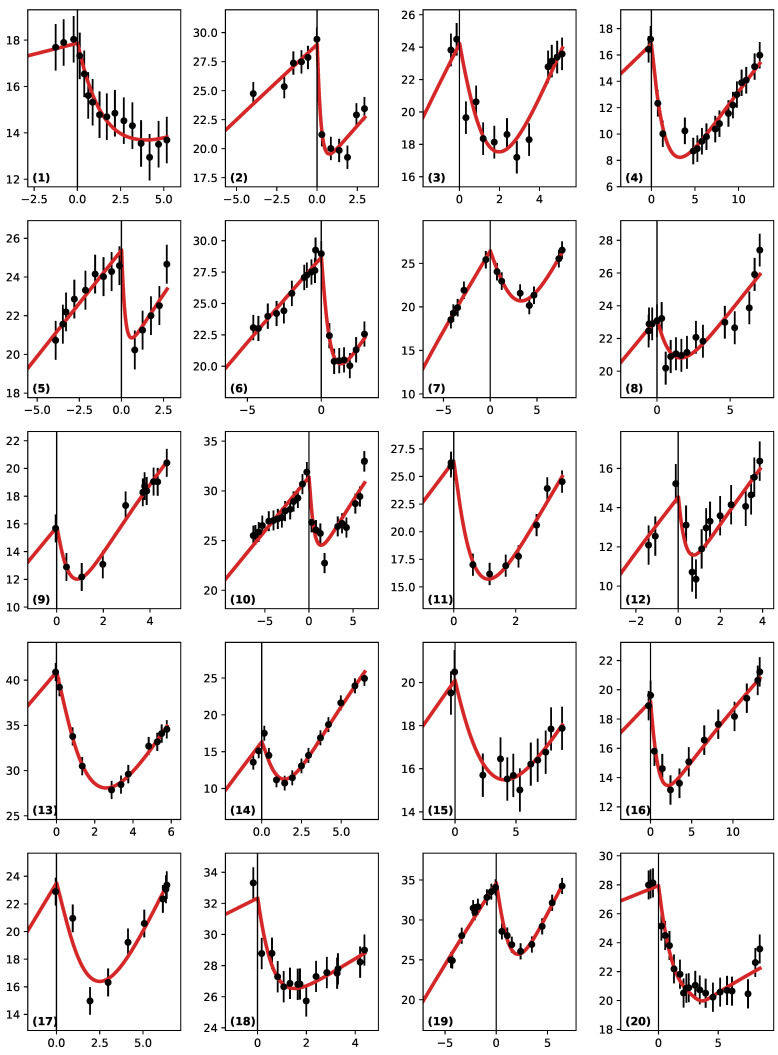
Comparison between the data points (in black) and our best fit models (red curve) for 20 patients. The abscissa represent time in years (with the origin at RT) and the ordinate of the tumor radius (in mm). Note that the scales are floating and span various ranges. The error bars on the measurements are 1 mm.

For each of our 43 patients, we performed the 5D minimization of the objective function in Equation (6). The minimal value of the $\chi^{2}$ function (called $\chi_{min}^{2}$) was reached for a set of parameters $\left(\hat{T},\hat{D},\hat{\kappa },{\hat{\kappa }}_{d},\hat{\tau }\right)$ that represented the “best fit" for each patient. Figure 2 shows the agreement between our best fit model and the data for a large number of patients with various medical follow-ups.

Our model allowed the reproduction of all of the different cases in a very satisfactory way for all 43 patients. For reasons of space, we present the results for a subset of 20 patients (the ones with the largest numbers of points). The 23 remaining fits are available in the Supplementary Material.

### 3.3. Tumor Age

An original aspect of this work is that we consider the age of the tumor (defined with respect to the time RT) as a free parameter, and we will now show that it is possible to get some information about this parameter.

Even when there is a substantial number of points before RT (which is rarely the case), one cannot simply linearly extrapolate back in time to determine the tumor birth date: There exists an invisible phase corresponding to the early development of the tumor that is below the detection level and, thus, not detectable [16,36]. In order to put a constraint on the tumor age *T*, we resort to the technique of the *profile likelihood* (see, e.g., [37]), which we will now explain.

The tumor age is *fixed* at some value *T*, and a minimization over the four remaining parameters is performed, giving a $\chi_{min}^{2}\left(T\right)$ value. The procedure is repeated for several *T* values. By shifting all of the values to have zero as the lowest value, one can reconstruct the profile-likelihood $\Delta \chi^{2}\left(T\right)$ of the tumor age. This profile can now be used to put a quantitative constraint on the *T* parameter. Indeed, it can be shown (e.g., [38]) that this function converges to a $\chi^{2}$ distribution with one degree of freedom so one can use its quantiles to get confidence level intervals. In particular, one obtains 95% confidence level intervals by thresholding the profile likelihood at 3.84.

We reconstructed the constraint on the tumor age (at RT) for all of our 20 selected patients (they are available in the Supplementary Material)and highlight some typical cases that show why the constraint depends crucially on the number of data points in the patients’ follow-ups (Figure 2).

- (a): Patient (6): This is a case where there are many points before RT and few during the regrowth phase.
- (b): Patient (14) is the inverse, with few points before RT, but the regrowth is strongly sampled.
- (c): Patient (13): No points before RT and a few during regrowth.

**Figure 3:**
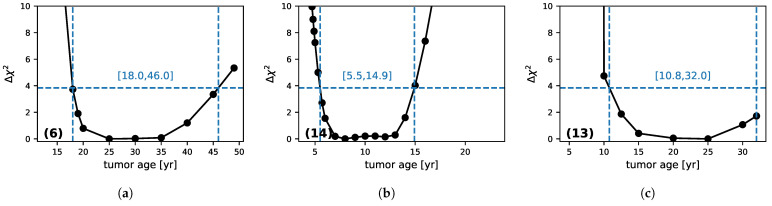
Constraints on the tumor age (at RT) with the profile-likelihood method. Results are shown for three patients (**a**–**c**) corresponding respectively to the data labelled (6), (14) and (13) on Figure 2. Considering the region below $\Delta \chi^{2}<3.84$ (the horizontal dashed line), one can reconstruct a 95% confidence level interval (shown by the dashed vertical lines and corresponding range values). The last value of the abscissa is the patient age.

The corresponding profile likelihoods are shown in Figure 3.

**Figure 4:**
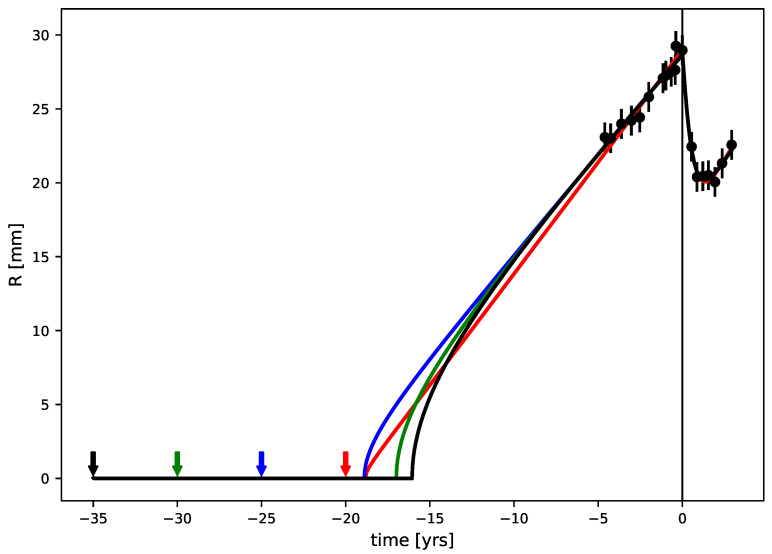
Full evolution of the radius of the four models corresponding to the bottom points ($\Delta \chi^{2}<1$) from Figure 3a. The origin time is fixed at RT, and the starting time of the tumor is indicated by colored arrows. All of the models give similar $\chi^{2}$ values with respect to the data.

For patient (a), one obtains both a minimum age constraint (18 years) and a maximum one (46 years). The points before RT fix both the radius at RT and the slope. The latter essentially constrains the product Dκ through the asymptotic speed ($v=2\sqrt{D\kappa }$). The invisible phase depends essentially on the proliferation rate κ and has a natural limit [16] because its duration cannot be smaller than the time at which the first point was measured. This fixes the lower limit of the tumor age. The upper limit comes from the fact that when the age increases, the proliferation rate gets smaller. However, for small κ, the evolution of the radius after the invisible phase is more and more curved. So, for a proliferation coefficient that is too small, the model is in disagreement with the data near RT and with the constraint on the linearity of the evolution at r=15 mm. This fixes the upper limit of the tumor age. Between these two limits, several models that correspond to several sets of parameters fit the data equally well. This is illustrated in Figure 4, where we plotted the four models corresponding to the four points (“bottom points”) with the lowest values of $\chi^{2}$ from Figure 3a (at 20, 25, 30, and 35 years), which all agree well with the data. One can see that the black model, which is the furthest from RT (35 years), has more curvature than the others and that the red one, which has the lowest age (20), corresponds to a very brief invisible phase. In this case, the silent phase is close to its minimum compatibility with the data points for this patient.

Patient (14) does not have much of a constraint before RT (Figure 2). However, the same type of constraint arises from the regrowth phase, which still follows the asymptotic limit, so we still obtain a full range of valid tumor ages.

Finally, although patient (13) has no points before RT and has points only at the beginning of the regrowth phase, one can still put a lower limit on the tumor age through the full fitting of the five-parameter model to the data. This demonstrates the potential of this method, which can still put some minimum bounds on the tumor age by exploiting the full information from the data.

**Figure 5:**
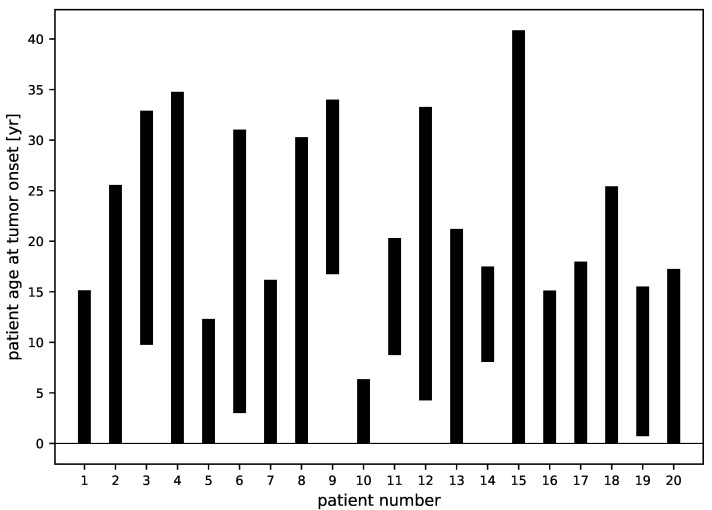
The 95% confidence level intervals of the ages of the patients at the onset of the tumors determined from the profile-likelihood analysis.

Using the profile-likelihood reconstruction of all 20 patients, we can study the ages of the patients at the birth of the tumors, which is calculated as the age of the patient at RT minus the age of the tumor at RT. We show in Figure 5 all 95% CL intervals obtained with this method. Although the constraints depend crucially on the data (size and sampling dates), they are consistent with the appearance of a DLGG at adolescence, as predicted in [16].

### 3.4. Tumor Characteristics

As already pointed out, the tumor age is a parameter that is very different from the other ones: It is only an unknown of the problem, which is why we treated it separately. The other four parameters ($D,\kappa ,\kappa_{d},\tau_{d}$) describe the DLGG’s evolution, but, for a given tumor age, they are strongly correlated.

**Figure 6:**
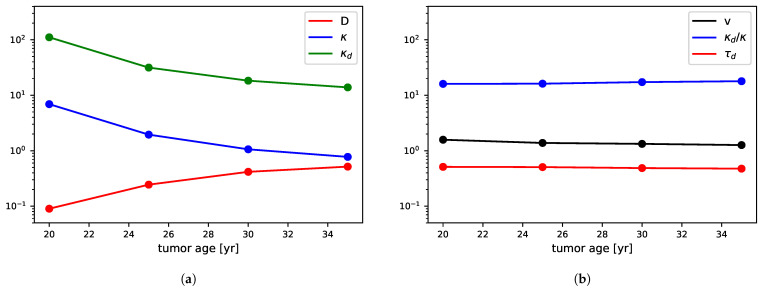
(**a**) Value of diffusion, proliferation, and death rate coefficients of the four models corresponding to the bottom points from Figure 3a ($\Delta \chi^{2}<1$). (**b**) The same for the transformed set of variables described in the text.

To illustrate this, let us consider again all of the models corresponding to the “bottom points” from Figure 3a ($\Delta \chi^{2}<1$). We show how their values depend on the tumor age; see Figure 6a. While all of the models are essentially equivalent in terms of $\chi^{2}$, the parameters vary considerably (in a correlated way), prohibiting any interesting individual constraints.

Inspired by Equation (17), we now use the following parameters instead: $v=2\sqrt{D\kappa },\frac{\kappa_{d}}{\kappa }$, and $\tau_{d}$. We show in Figure 6b that they are indeed more stable in the valid age range, so the variables are now uncorrelated.

**Figure 7:**
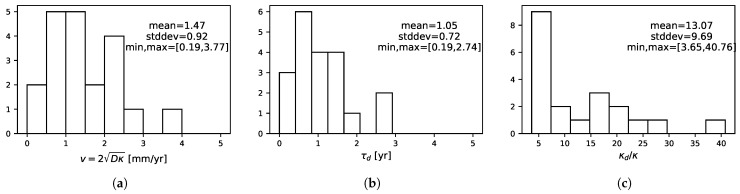
Histograms of the best fit parameters for the velocity parameter (**a**), death characteristic time (**b**), and proliferation ratio (**c**) for our selection of 20 patients.

We then study if there are some common features among our patients. Figure 7 shows the histograms of the measured characteristics.

The measured velocities are consistent with the DLGG values for velocities, which are in the typical [1,4]-mm/yr range [39]. The characteristic death rate times are about $\tau_{d}=1.0\pm 0.7\;\mathrm{yr}$. The ratio between the death and proliferation rates after RT is large—typically above 5, but with a wide distribution, with values that can go up to 40.

We can also check if there are some correlations between the evolutions before and after RT. From the best fit models, we compute the following observables: $V_{-}=\frac{dr_{mod}}{dt}\left(t_{r}^{-}\right)$), which is the slope just before RT, $t_{min}$, which is the time at which the minimum is reached, $\Delta R=r\left(t_{r}\right)-r\left(t_{min}\right)$, which is the amplitude decrease at the minimum point, and ΔT, which is the time interval after RT when the radius comes back to its value at the time of RT.

In the dataset, we measure the following correlation coefficients:

(18) $$\begin{array}{cc} <V_{-},\Delta R> & =+0.42\\ <V_{-},t_{min}> & =-0.27\\ <V_{-},\Delta T> & =-0.63. \end{array}$$

From the (approximate) radius evolution in Equation (17), we have

(19) $$\begin{array}{ccc} \Delta R & ≃ & v\tau_{d}\;\left(\frac{\kappa_{d}}{\kappa }-\ln\frac{\kappa_{d}}{\kappa }-1\right)\\ t_{min} & ≃ & \tau_{d}\ln\frac{\kappa_{d}}{\kappa }\\ \Delta T & ≃ & \tau_{d}\frac{\kappa_{d}}{\kappa } \end{array}$$

where *v* is the theoretical value $v=2\sqrt{D.\kappa }$.

From these expressions, we expect some correlation between *v* and ΔR, an anti-correlation between *v* and $t_{min}$ through κ (but moderate because of the logarithm), and a stronger one with ΔT. This corresponds exactly to what is observed in the dataset. We can conclude that our model correctly reproduces the correlations observed in the data before and after RT.

## 4. Discussion

DLGGs are tumors that always turn into a more aggressive form after years of indolent growth [40]. They are also resistant to RT, since they systematically recur after the end of the treatment. Modeling their dynamics with and without treatment can lead to a better understanding of their evolution and their resistance to treatments.

Here, we complement a classical diffusion–proliferation model (that has already proved its usefulness for the evolution of DLGGs) with a model of the RT effect as a simple, time-dependent, and spatially structured death term. The spatial dependency of the death term means that cells at the border of the tumor are killed more than cells at the center of the tumor. The time dependency of the death term translates into a net proliferation term (proliferation minus death) that is time-dependent: Before RT, the net proliferation is positive, and then negative during a certain time interval after RT. When the death coefficient is smaller than the proliferation parameter κ, the net proliferation term is positive again, and the tumor resumes its growth at the same rate as before RT.

The first qualitative feature that our model reproduces—without RT—is the fact that proliferating cells are situated at the border of the tumor. This spatial effect has been observed in human tissue from DLGGs; by analyzing tissue samples from stereotaxic biopsies, it has been shown that cycling cells (or proliferating cells) are situated at the border of the tumor [41]. Even if DLGGs do not have a necrotic (and even hypoxic) center as higher-grade gliomas do, the cell density is still higher than normal. It is thus possible that some regions of sub-optimal oxygen concentration develop at the center of the tumor, thus reducing proliferation and triggering the transformation of cells into quiescent ones. We will see later that this spatial organization, which our model reproduces well, is crucial in the modeling of the action of RT.

Another important point is the modeling of the RT effect. Our death term due to RT is designed to preferentially target proliferating cells (see Figure 1), since it is well known that proliferating cells are the most sensitive to irradiation and die via mitotic catastrophe in particular [42]. However, our death term is also time-dependent (with a characteristic time that can be from months to a few years depending on the patients), and we will now discuss this point. Although it is certainly a complicated effect that varies among patients, we argue that our choice of a time-dependent death rate is biologically realistic. Tumor irradiation induces both direct and indirect effects that could lead to tumor cell death. Direct effects are the result of radiation-induced DNA damage in cancer cells that are too important to be repaired (double-strand breaks in the DNA molecules). However, RT can also induce indirect damage to DNA (via reactive oxygen species) and to the tumor microenvironment, such as in the vasculature. It can also trigger an immune response that can contribute to the tumor growth control [43,44]. Usually, damaged cells try to repair the damage and can even try to go through several mitoses before triggering their death. All of these process can take some time, and this is why the response to RT can be prolonged in time. We decided to model this delayed effect by defining a characteristic time in the death term. The choice of the exponential function for the death term is the simplest way to introduce a characteristic time. However, it could also be justified in an other way: The linear quadratic model stipulates that the survival time is an exponential function of the dose received (for a review on the linear quadratic model, see [45]). However, the efficacy of a given dose was measured with cell culture in 2D. In a real tumor, it is possible that the efficacy of a dose depends on the microenvironment. It is a well-known fact that hypoxic cells (at the center of tumors) are more resistant to RT than normoxic cells [46]. Actually, this constitutes an important limitation to the use of RT. So, for a given dose that is received, the radiation could have a smaller effect for cells that are closer to the poorly oxygenated center than at the well-oxygenated border, leading to a larger survival rate. It is also well known that radiations kill proliferating cells, which cannot repair the DNA damage when trying to undergo mitosis. Just after RT, the more damaged cells begin to die, and quiescent cells are now at the border and turn into proliferating ones; thus, they also die, but with a death rate that is lower than that in the first layer. Thus, cells would die layer by layer, from the outside inwards. This process would justify our death rate that is exponentially decreasing with time. Biologically, this process is realistic; it has actually been observed in vitro and modeled with a cellular automaton for spheroids in [47,48].

If we compare our model with the few models of the effects of RT on DLGGs, we can note that our model is the one that can reproduce the most biological characteristics of DLGGs without and with RT. First, the model displays a space dependency (and this is not the case for the model in [17]) that allows one to find that proliferating cells are at the border of the tumor and that the velocity of the evolution of the tumor radius is linear. Second, our model also reproduces the most striking feature of the evolution of DLGGs under RT, which is the fact that the tumor radius continues to decrease even after the end of the treatment over a few months to almost ten years, depending on the patient, before the tumor systematically recurs and starts to grow again. This behavior is not accounted for by the model in [23], where the tumor starts to grow again just after the end of the treatment. Third, when compared to large amounts of high-quality clinical data (patient follow-ups with tumor radius measurements for several time points), our time-dependent death rate model could reproduce the exponential shape that is visible in the experimental data: a sharp decline in the first period, followed by a slower decay, and an almost linear regrowth. With simple analytical considerations, we show that with a constant death term, as in the models in [18,19], the decrease in the radius can only be linear (at best) and cannot lead to any exponential-like decrease.

We took care not to introduce too many parameters in our model in order to continue to allow the flexibility to describe all of the data. The tumor evolution with RT is only described by four parameters, with two being the natural evolution ones (proliferation and diffusion) and two for the RT effect (death rate and characteristic time). An original aspect of this work is that we also considered the (unknown) tumor age as a free parameter that could, therefore, be constrained by the data.

With this five-parameter model, the data of the temporal evolution of the tumor radii for 43 patients were automatically fitted, and excellent results were given. We selected 20 patients with more than 10 data points (the fits for the other 23 patients are available in the Supplementary Material), and for each patient of this series, by scanning the possible ages of the tumor—from 0 to the patient’s age—we could infer the possible age range of the patient at the onset of the tumor. We found that the age at the onset of the tumor that was compatible with most of the patients was around 15 years-old. This finding confirms previous research [16], where, from the data on the velocity and one measure of the tumor radius at a given time and going back in time with a model, the conclusion was that patients were most likely to be in their late teenage years at the onset of the tumor.

This age at the onset of the tumor depends on the initial conditions; if the simulation starts from a small clump of cells, the time needed to form that clump is not counted, and the age of the tumor is underestimated. Moreover, the choice of the size of the clump would have been subjective. We chose to start the simulations from one cell. We also assumed that the proliferation and diffusion coefficients were constant all along the tumor’s evolution. This is a strong assumption that may not be correct. It is indeed possible that the first cell only proliferates and forms a small clump of cells before diffusion takes place. On the other hand, since we do not have any clue about what happened at the beginning of the evolution, and since even when discovered early, DLGGs seem to grow the in same way as larger tumors (associating proliferation and diffusion), we decided that the simplest way to choose the initial conditions was to start with one unique cell and the same proliferation and diffusion coefficient.

For the population of 20 selected patients, we also measured a characteristic RT time $\tau_{d}$ of around 1 year and a ratio of the maximal death coefficient to the proliferation coefficient $\kappa_{d}/\kappa$ that was always larger than 5. The fact that this ratio has a value larger than 1 is an important biological result; it means that just after RT, a large quantity of cells is killed just after RT, and does not have time to go through mitosis. In these cells, the damage due to RT may have been so important that the cells triggered the apoptosis program immediately without even trying to perform mitosis.

With the group of the 20 selected patients, we could also highlight good correlations between the velocity before RT, $V^{-}$, and both the gain of lifetime ΔT and ΔR (the maximum decrease in the radius). A simple analysis allowed us to understand these correlations.

Now that we have shown that our model is the most efficient model for describing the effects of RT on DLGGs, in our future work, we plan to use the results of this study to predict whether patients have an early or late regrowing tumor with only one or two data points after RT. This prediction could be used to improve follow-ups with patients by adapting the frequency of MRI scans.

## Figures and Tables

**Figure A1:**
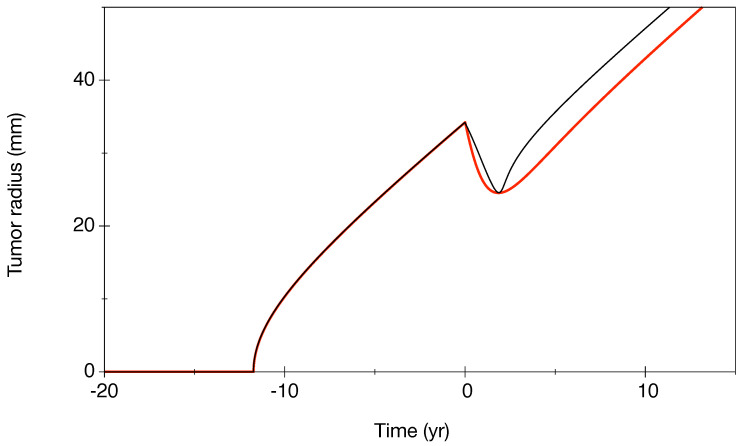
Temporal evolution of the tumor radius versus time for two models: one with a constant death rate (black line) and a fraction of the cell density damaged at the time of RT and a model with a time-dependent death rate (red line). The origin of the time was set to the time of RT. The parameters for the constant death rate model: κ=1 yr${}^{-1}$, D=1 mm${}^{2}$ yr${}^{-1}$, $\kappa_{d}=3.3$ yr${}^{-1}$, x=0.0053. The parameters for the time-dependent death rate model: κ=1 yr${}^{-1}$, D=1 mm${}^{2}$ yr${}^{-1}$, $\kappa_{D}=8.5$ yr${}^{-1}$,$\tau_{d}=0.9$ yr.

## Supplementary Materials

The following are available online at https://www.mdpi.com/article/10.3390/jpm11080818/s1.

## Appendix A

In this appendix, we compare the evolution of the tumor radius obtained with our model with that of a constant death rate model with two populations, which was inspired by Perez’ and Ribba’s work [17,19]. In this model, RT damages a fraction of the cell population, and this damaged population evolves differently from the undamaged one; the undamaged cells continue to proliferate and diffuse at the same rate as before, whereas the damaged population stops proliferating and dies progressively (but still diffuses normally). A model of this type is interesting because it is biologically realistic and it accounts for the delay in the regrowth of the radius after RT. All of the parameters are constant and, aside from the two parameters κ and *D*, which describe the natural evolution of the tumor, two parameters are needed for RT: the fraction of the cell population that has been damaged, *x*, and the death rate of this population, $\kappa_{d}$.

For $t<t_{r}$, the equation describing the evolution of the cell population is the same as Equation (1):

$$\frac{\partial \rho }{\partial t}=D\Delta \rho +\kappa \rho \left(1-\rho \right)$$

For $t=t_{r}$, two populations are created: the damaged one, $\rho_{d}\left(t_{r}\right)=x\rho \left(t_{r}\right)$, and the undamaged one, $\rho_{nd}\left(t_{r}\right)=\left(1-x\right)\rho_{nd}\left(t_{r}\right)$. After RT ($t>t_{r})$:

$$\frac{\partial \rho_{nd}}{\partial t}=D\Delta \rho_{nd}+\kappa \rho_{nd}\left(1-\rho \right)$$

for the undamaged population, and:

$$\frac{\partial \rho_{d}}{\partial t}=D\Delta \rho_{d}-\kappa_{D}\rho_{d}\left(1-\rho \right)$$

with $\rho =\rho_{d}+\rho_{nd}$.

This model can reproduce the delay in the regrowth after RT. However, as shown in Section 3.1, the decrease in the radius is, at best, linear, which is clear in Figure 2, which is not in agreement with the clinical data, where a steep decrease is generally observed first, followed by some milder phases. In Figure A1, the time of RT was set to 0, and the RT parameters of the two models were chosen so that the minimum of the evolution of the radius was the same. The linear decrease obtained with a two-population model after RT can be compared to the exponential decay obtained with our one-population model. In the regrowth phase, the evolution of the radius with the two-population model displays a strong curvature that is reminiscent of the beginning of the visible evolution of the radius.
