# Supplementary material for: The Effect of Radiotherapy on Diffuse Low-Grade Gliomas Evolution: Confronting Theory with Clinical Data
Source: J Pers Med. 2021 Aug 21;11(8):818. doi: 10.3390/jpm11080818 (PMC8401413; doi:10.3390/jpm11080818)
Supplement: Supplementary file 1 [file jpm-11-00818-s001.zip › jpm-1309226-SI.pdf]

Supplementary Material

Radiotherapy Effects on Diffuse Low-Grade Gliomas: Confronting Theory with Clinical Data

L. Adenis, S. Plaszczynski, B. Grammaticos, J. Pallud and M. Badoual *Université Paris-Saclay, CNRS/IN2P3, IJCLab, 91405 Orsay, France* and *Université de Paris, IJCLab, 91405 Orsay France*

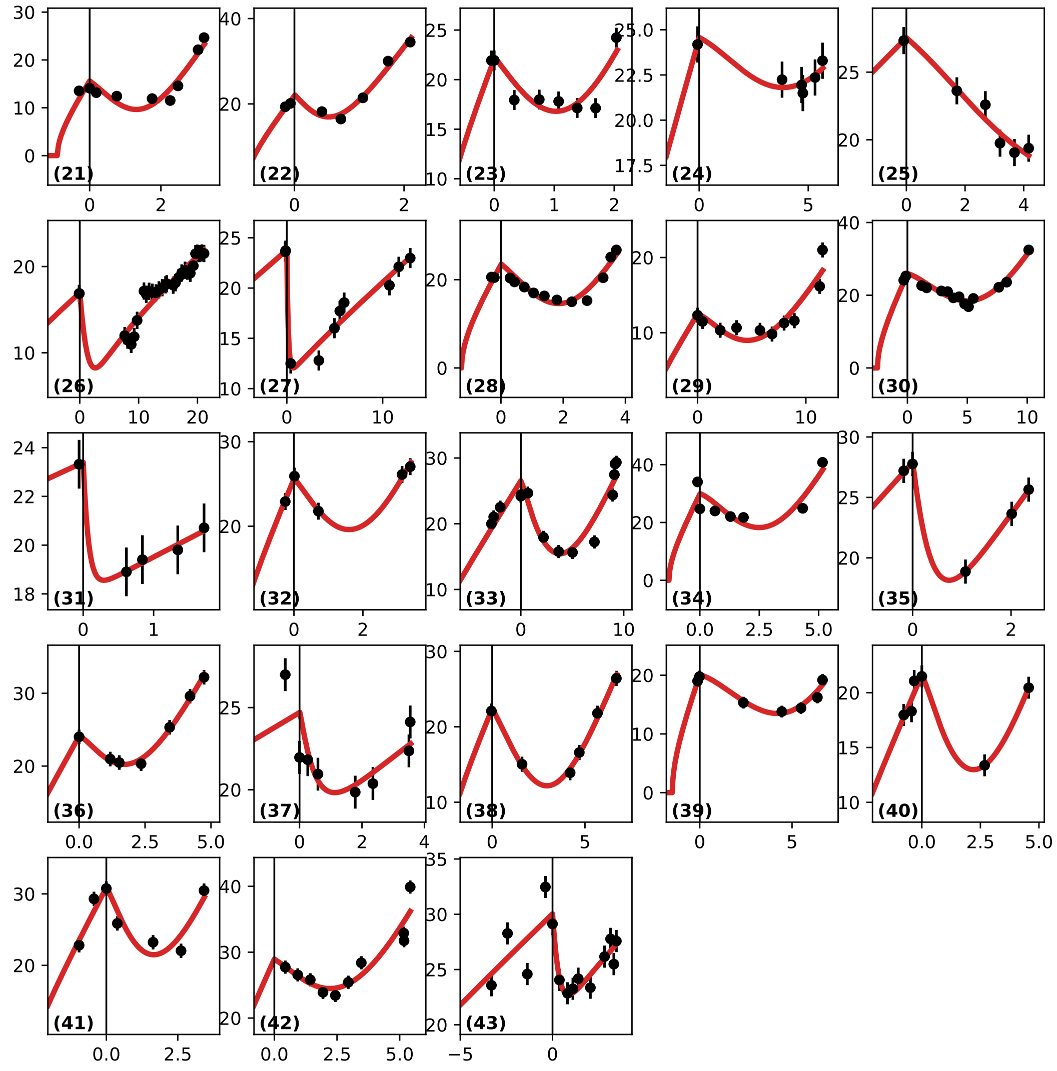

Supplementary Figure S1: Comparison between the data points (in black) and our best fit models (red curve) for the 23 patients that are not included in Figure 2. As in Figure 2, the abscissa represent time in years (with the origin at RT) and the ordinate the tumor radius (in mm). Note the scales are floating and span various ranges. The errors bars on the measurements are of 1 mm.

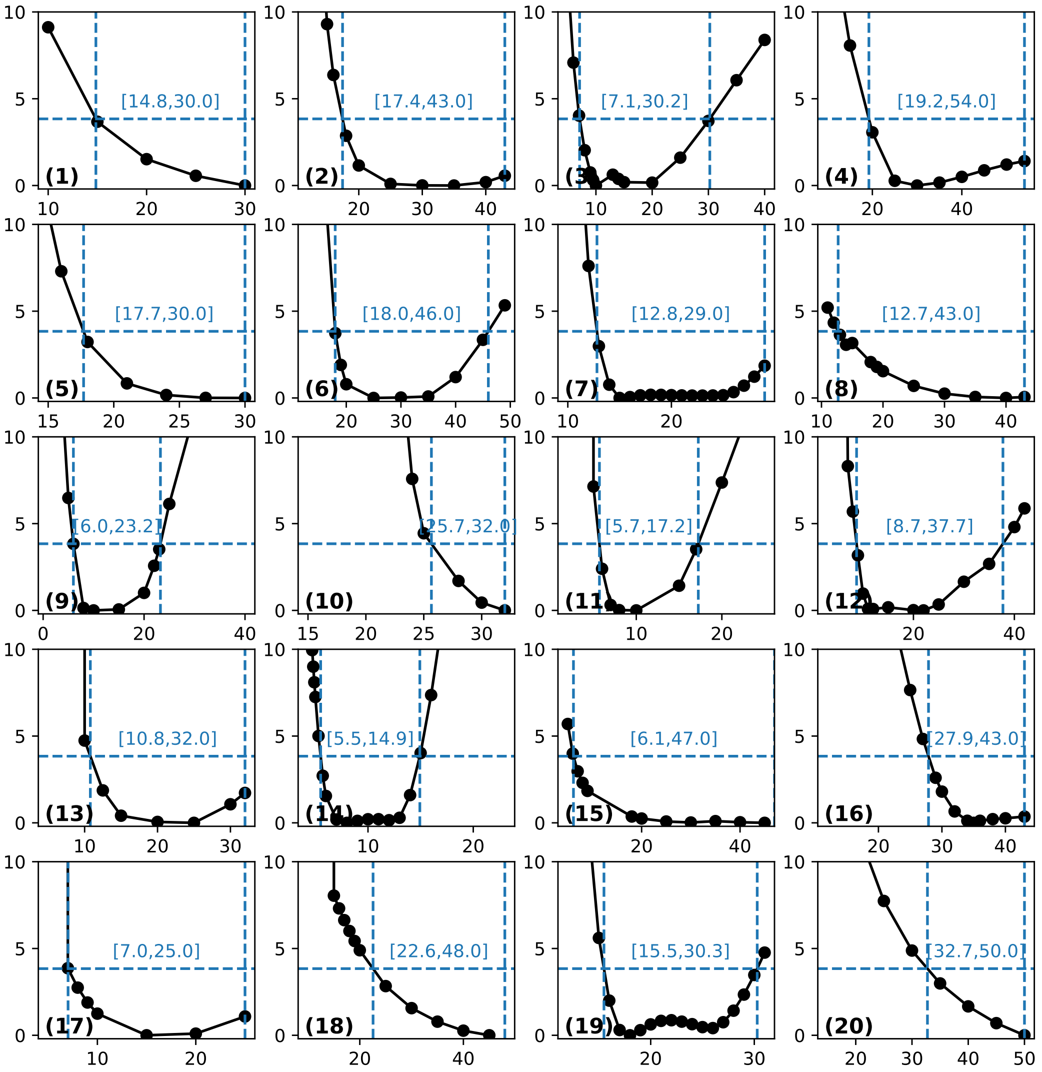

Supplementary Figure S2 : Constraints on the tumor age (at RT) with the profile-likelihood method. Results are shown for the 20 patients selected in Figure 2. Considering the region below $\Delta\chi^2 < 3.84$ (the horizontal dashed line), one can reconstruct a 95% confidence level interval (shown by the dashed vertical lines and corresponding range values). The last value of the abscissa is the patient age.
